# Samarium Monosulfide (SmS): Reviewing Properties and Applications

**DOI:** 10.3390/ma10080953

**Published:** 2017-08-16

**Authors:** Andreas Sousanis, Philippe F. Smet, Dirk Poelman

**Affiliations:** Lumilab, Department of Solid State Sciences, Ghent University, Krijgslaan 281/S1, 9000 Gent, Belgium; andreas.sousanis@ugent.be (A.S.); philippe.smet@ugent.be (P.F.S.)

**Keywords:** SmS, switchable material, thin films, magnetic properties, optical properties, deposition techniques

## Abstract

In this review, we give an overview of the properties and applications of samarium monosulfide, SmS, which has gained considerable interest as a switchable material. It shows a pressure-induced phase transition from the semiconducting to the metallic state by polishing, and it switches back to the semiconducting state by heating. The material also shows a magnetic transition, from the paramagnetic state to an antiferromagnetically ordered state. The switching behavior between the semiconducting and metallic states could be exploited in several applications, such as high density optical storage and memory materials, thermovoltaic devices, infrared sensors and more. We discuss the electronic, optical and magnetic properties of SmS, its switching behavior, as well as the thin film deposition techniques which have been used, such as e-beam evaporation and sputtering. Moreover, applications and possible ideas for future work on this material are presented. Our scope is to present the properties of SmS, which were mainly measured in bulk crystals, while at the same time we describe the possible deposition methods that will push the study of SmS to nanoscale dimensions, opening an intriguing range of applications for low-dimensional, pressure-induced semiconductor–metal transition compounds.

Switchable materials are a specific category of compounds presenting changes between two distinct states, with a simultaneous change of material properties. Several kinds of switchable materials showing a semiconductor–metal transition are known. In phase change materials such as AgInSbTe and Ge_2_Sb_2_Te_5_, there is a reversible transition between an amorphous and a crystalline phase, by using either optical or electrical pulses, changing both the structure of the initial material and the corresponding optical and electrical properties. These materials can be used in non-volatile memory devices, exploiting characteristics from flash memories and dynamic random access memories, respectively [1,2,3]. Vanadium dioxide (VO_2_) is another example of a switchable material, which could be exploited in several applications, such as thermochromic windows [4,5,6,7]. In this case, the transition is related to a thermally induced change between the rutile and tetragonal phase, which is accompanied by changes in its properties. Samarium monosulfide (SmS) is known for its pressure-induced semiconductor–metal transition, leading to a plethora of exciting physical properties. In this review, we will discuss some of the most important results about SmS, showing possibilities to use it as a material for a new kind of non-volatile memory, which could exploit the pressure-induced transition of SmS. This kind of application requires new research on material properties at low dimensions. This review first describes bulk material properties but also attempts to stimulate further exploitation and study of SmS thin and ultra-thin films.

## 1. Introduction

**Figure 1:**
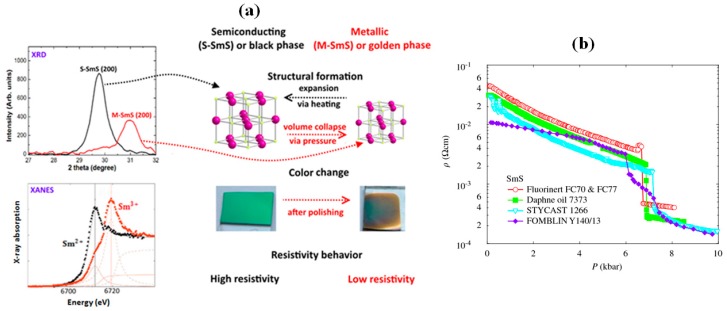
(**a**) Basic properties of SmS. The XRD pattern (upper left) demonstrates the shift of the (200) diffraction peak, when S-SmS (semiconducting, black curve) switches to M-SmS (metallic). The lower left graph shows the corresponding valence change, as evident from a XANES measurement. In the S-SmS or black state the material shows a NaCl structure with a black-blue color and a high resistivity. The M-SmS or golden state has the same structure with a volume collapse. The color changes to gold and the resistivity drops up to four orders of magnitude. For the unit cell of SmS: purple spheres, Sm; yellow spheres, S. (**b**) Resistivity drop of SmS single crystal. Four different pressure transmitting media are indicated in the graph. (Reprinted with permission from [26], [27] and [24], respectively.)

SmS is an intriguing material, which shows a pressure-induced transition from the semiconducting to the metallic state [8,9,10]. The transition is also called the black–golden transition, referring to the typical color of the SmS in the semiconducting and metallic state, respectively. Generally, the transition appears at about 6.5 kbar, at room temperature [8,11,12,13]. The importance and uniqueness of this material is based on its switching characteristics, as other switchable materials require proper heating/cooling cycles, such as VO_2_, or only show a metal–insulator transition at low temperatures, due to the splitting of the f-band in strongly correlated systems. As we will discuss further, the moderate pressure required for switching, the possibility to modify the material to make the transition reversible, and finally the fact that the transition takes place at room temperature, all make SmS a highly interesting material from the applications point of view. From the first investigations by Suryanarayanan et al. [14] to more recent work of Rogers et al. [15] the optical spectra of SmS have been studied in order to gain a deeper understanding of the absorption peaks and the nature of the transition. SmS possesses a NaCl (fcc/rock salt) structure [13,15], which remains unaltered after transition, as seen in Figure 1a. The isostructural transition contrasts with other phase change materials, where changes in resistivity and optical properties are caused by a change in crystallographic phase. The process that takes place in SmS is a volume collapse, with a decrease in unit cell volume by approximately 13.5% [16]. The semiconducting phase can be restored by thermal annealing. By alloying with europium (Eu) or gadolinium (Gd), the pressure at which the phase transition occurs can be changed [17,18]. The Mott criterion gives the concentration of electrons required for the electronic transition to occur, independent of the method to induce the transition, such as light, heat or pressure [10,19,20]. It has been found that, in the case of a laser-induced non-thermal semiconductor–metal transition, the minimum density of electrons upon the transition should approach the electron density of the metallic state [20,21]. In Figure 1a (upper left graph), we see the main X-ray diffraction (XRD) peak of a semiconducting SmS thin film and its shift to larger angles in the pressure induced metallic phase [15]. This shift indicates a decrease in lattice constant, which can be used as a probe for the transition to the metallic state. Moreover, theoretical models such as the theory of excitonic instability can predict and explain this transition, as presented below.

The semiconducting–metallic transition of SmS is driven by the variable oxidation state of Sm. Samarium has two possible ground state configurations in SmS, a nonmagnetic Sm^2+^ with 4f^6^(^7^F_0_) configuration and a magnetic Sm^3+^ with 4f^5^(^6^H_5/2_) configuration [22]. It has to be noted that an intermediate valence state is also possible. Other mixed valence Sm compounds are SmSe, SmTe and SmB_6_ [12,23]. We can observe the changes in valence state of SmS in X-ray absorption near edge structure (XANES) measurements, as shown in Figure 1a (lower left graph). The Sm valence state changes from a predominantly 2+ state (black, semiconducting state) to a mainly 3+ state (golden, metallic state), with a remaining shoulder at the position of Sm^2+^, related to the existence of a mixed valence state or to the presence of non-switched parts in the thin film. In order to quantify these results, least-squares fitting was used, showing an intermediate valence, even above 2 GPa, where the magnetic order appears. More details on this behavior are given below. In addition, computations showed that Sm does not acquire its fully trivalent state even at a pressure higher than 6 GPa. The lattice constant decreases as the valence state increases, due to the large difference in ionic radius for Sm^2+^ and Sm^3+^ upon the transition to the metallic state.

In Figure 1b, we can see the behavior of the SmS single crystal resistivity as a function of hydrostatic pressure. In general, the resistivity drop occurs at close to 6.5 kbar (at room temperature), but there are small differences depending on the experimental measurement conditions [24]. The resistivity as a function of pressure can be expressed as: $\rho_{P}=\rho_{0}e^{-\left(\alpha \right)P/kT}$, where $\alpha =d(\Delta E_{g})/dP$ is the pressure dependent gap between the f-d bands. In the case of a continuous transition of SmS, the ambient pressure energy gap ΔE_g_ can be found from the $\rho_{a}/\rho_{h}$, where ρ shows the resistivity at ambient pressure, while $\rho_{h}$ represents the resistivity at high pressure, since $\rho_{h}=\rho_{a}e^{-\Delta E_{g}/kT}$. The energy gap between the f and d band goes to zero, before the transition [25].

Up to this point, we briefly mentioned the basic properties and switching characteristics of SmS, as well as ways to monitor these properties. In the next sections, we will describe the pressure related electronic, optical and magnetic properties of SmS in a more detailed way, as they were typically studied in bulk crystals. While interesting in itself, the utilization of these properties in real-world applications requires high quality SmS thin films. As the deposition of stoichiometric SmS films with the correct phase and Sm valence state is far from obvious, part of this review will be devoted to describing the different methods to synthesize SmS thin films.

## 2. Properties of SmS

### 2.1. Energy Level Diagram

**Figure 2:**
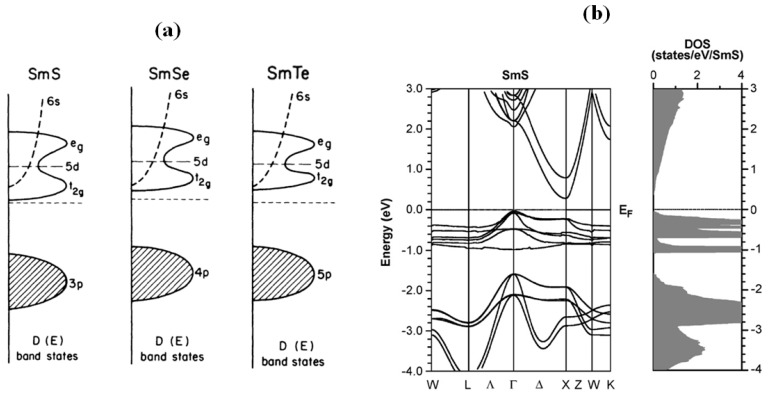
(**a**) Band structures for semiconducting monochalcogenides SmS, SmSe and SmTe, where D(E) is the density of states. For these cases, the gap between occupied and unoccupied states is different according to Batlogg, with the 4f^5^ state fully occupied; (**b**) Band structure from computational work, in the semiconducting state following the LSDA+U (Coulomb repulsion energy of Sm 4f) method with the contribution of spin-orbit coupling. (Reprinted with permission from [30] and [34], respectively.)

Electronic structure and pressure related properties of SmS were reviewed in 1993 by Wachter [28], essentially on bulk SmS. Initially, the basic properties, such as piezoresistivity and optical changes have been studied, while aspects of the Kondo-like behavior and laser-induced transition properties have been investigated over the last years. The main electronic difference between SmS and other chalcogenides is the fact that the gap between the 4f^6^ ground state of the Sm^2+^ ion and the 4f^5^5d(t_2g_) degenerate state is smaller in comparison with other similar materials [12,15,29]. In Figure 2a, the band structure of three Sm chalcogenides is shown, as calculated by Batlogg et al. [30]. Recently, it has been reported that the energy between the p-character of the valence band and the 4f states of the samarium ions is constant [31]. By pressurizing SmS, the 5d degenerate state moves toward the 4f^6^ ground state of the Sm^2+^ ion, because the increase in pressure leads to an increase of the energy splitting of the doubly degenerate (e_g_) and triply degenerate (t_2g_) 5d states [32,33]. In Figure 2b, we can observe that the band structure separates into three parts, as shown in the density of states (DOS). The SmS 5d conduction band is 0.25 eV above the Fermi level, the rather localized Sm 4f levels are situated between 0 and −1 eV and the highly dispersive S 3p valence band is located below −1.6 eV [34]. There is an indirect gap between the Γ point of the valence band and the bottom of the conduction band at the X point, equal to 0.25 eV. Moreover, the direct gap is equal to 0.5 eV.

### 2.2. Electrical and Optical Properties

**Figure 3:**
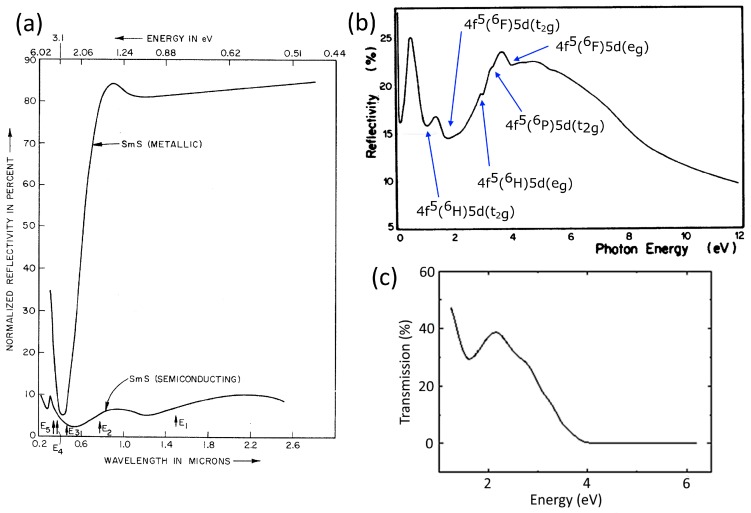
(**a**) SmS reflectivity as a function of wavelength in both metallic and semiconducting phase (SmS single crystal); (**b**) Reflectivity (SmS single crystal) of the semiconducting phase. The final levels of the transitions are labelled in the graph, while all of the transitions start from 4f^6^(^7^F_0_); (**c**) Transmission of a 100 nm thin film S-SmS. ((a) and (b) adapted from [39] and [30], respectively, with permission.)

Here, the behavior of the dielectric function in both semiconducting and metallic states is presented. There are five prominent optical transitions in semiconducting SmS between 0.8 and 4 eV, as observed by UV/VIS spectroscopy [15,35] and angle-resolved photo-emission spectroscopy (ARPES) [32]. The first transition with a maximum at 0.8 eV (6650 cm^−1^) is related to 4f^6^(^7^F_0_)-4f^5^(^6^H)5d (t_2g_); the second transition, located at 1.6 eV (13,215 cm^−1^), originates from 4f^6^(^7^F_0_)-4f^5^(^6^F)5d (t_2g_); and the third transition at 3.1 eV (25,000 cm^−1^) corresponds to the 4f^6^(^7^F_0_)-4f^5^(^6^H)5d (e_g_) transition. The other two transitions take place from 4f^6^(^7^F_0_) to 4f^5^(^6^P)5d(t_2g_) and 4f^5^(^6^F)5d(e_g_) and appear at about 3.6 eV and 4.0 eV, respectively (see Figure 3b). The basic knowledge about the optical properties of SmS comes from Batlogg et al., who studied the optical and electrical behavior of the semiconducting and metallic state. As we have already demonstrated, the cubic crystal field splits the 5d band in two sub-bands. In the metallic phase, the peak in the imaginary part of the dielectric function at around 5.5 eV is related to the transition from the 3p^6^ anion valence band of Sm ions to empty states above the Fermi level. Additionally, a steep kink at about 4.5 eV stems from transitions from the 4f^5^(^6^H_5/2_) state of Sm^3+^ to empty states above the Fermi level. In the visible range of the metallic phase, a valley is seen at 3.1 eV, due to the presence of free electrons (Drude model). The golden color of the metallic state is due to the reflectivity drop at around 0.45 μm (Figure 3a). This valley of the reflectivity at shorter wavelength is related the plasma frequency of the metallic SmS in the UV regime, with the resulting sharp dip at 0.45 μm coming from inter-band (bound electron) transitions [15,36,37,38,39].

Metallic SmS can be stable on the surface of a sample and can be obtained either by polishing, pressurizing or by scratching the surface of a semiconducting SmS sample. Nevertheless, a part of SmS can probably remain unaltered, in its semiconducting state, as switching can occur on a grain-to-grain basis [15]. Reflection measurements can be used to show that an amount *x* of SmS remains unaltered, in the semiconducting state. As a result, the reflectivity of the sample (with reflectivity R_(SmS)mix_) is the sum of the two fractions (with reflectivity R_M-SmS_ and R_S-SmS_) that simultaneously exist within the sample. In order to calculate the reflectivity of the metallic state, we can use: R_M-SmS_ = 1.18(R_(SmS)mix_ − *x*R_-SmS_) [28].

Very recently, some investigations have shown the existence of a pseudo-gap, before the final metallic behavior is reached, upon application of pressure. This effect has been studied in detail [40], by measuring the optical reflectivity under pressure, in both the middle and far infrared region of the electromagnetic spectrum. It was observed that the black–golden transition takes place when the gap size gets almost equal to the excitonic binding energy before it entirely closes to achieve the full metallic state. As a result, this transition essentially stems from an excitonic instability. The energy gap at a pressure of 0.65 GPa was measured to be 45 ± 20 meV, with an excitonic binding energy equal to 60 meV. Upon the semiconductor–metal transition, the electrons are mixed in the 5d conduction band with the 4f band of the divalent ion, with a transformation from the divalent to trivalent state of Sm ions. As the trivalent state has a smaller ionic radius, the lattice constant reduces, providing a further lowering in energy of the lowest 5d band, below the initial 4f state, leading to the valence change from mainly Sm^2+^ to mainly Sm^3+^ [40].

An intermediate valence state is determined by the exact position of the Fermi level in the gap and especially its location in comparison with a virtual localized state, which stems from the 4f^6^ level of the Sm^2+^ ground state. Above this virtual state, one obtains the divalent state, while a trivalent state occurs below it. When the Fermi energy is pinned to this state, we obtain an intermediate or a mixed-valence state, which could be explained by two possible mechanisms. The first one is related to a spatial mixture of valence states, in the case both divalent and trivalent ions are present in the material, providing a mixing and disorder on the atomic scale. The second interpretation comes from a temporal mixture, when every Sm ion temporally fluctuates between the two possible valence states. In practice, the second explanation is more probable for several reasons, such as the prohibitive strain energy of the spatial mixing [12,41].

### 2.3. Resistivity Drop and Structural Properties

**Figure 4:**
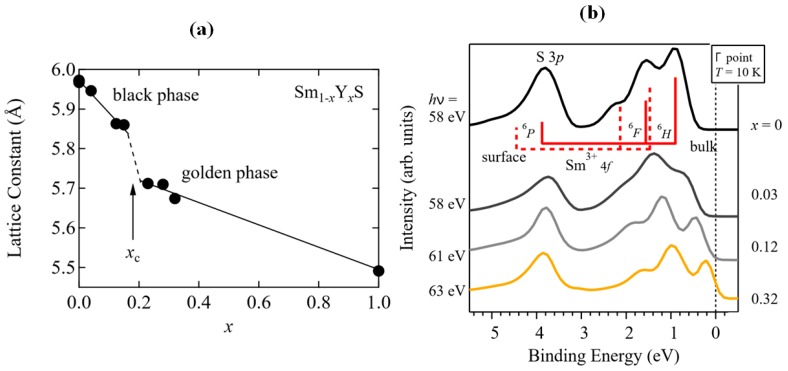
(**a**) Yttrium concentration dependence of the lattice constant of the SmS-based single crystal; (**b**) The density of states at the Γ point of the Brillouin zone of the SmS-based material. The photon energies and concentrations of Y are placed on the left and the right side of the graph, respectively. (Reprinted with permission from [42].)

SmS single crystals revert to the semiconducting state upon the release of the pressure, used for inducing the metallic state [12]. SmS thin films do not necessarily show this reversible behavior, as tensile pressure or annealing in vacuum is necessary to provide the back switching to the initial semiconducting state. Recently, Imura et al. [42] studied the electronic structure of Sm_1−*x*_Y*_x_*S (*x* = 0, 0.03, 0.12, or 0.32) single crystals by using ARPES in order to investigate the black–golden phase transition. By substitution of Sm by Y, the gap closes and the metallic state can be stabilized at room temperature (Figure 4a). The stabilization of the metallic state at ambient pressure occurs around a critical Y concentration of *x* = 0.2, due to the presence of intrinsic stress. The closing of the gap is visible in the ARPES spectra (Figure 4b) for increasing Y concentration. The solid and broken lines indicate the multiplet structure of the Sm 4f^5^ states (^6^H, ^6^F, ^6^P), where a discrimination is made between bulk and surface states. A similar effect can be obtained by using gadolinium instead of yttrium as an alloying element. Sharenkova et al. [43], studied Sm_1−*x*_Gd*_x_*S single crystals prepared by directed crystallization from the melt. Based on XRD measurements before and after the valence transition, it was substantiated that the decrease of scattering regions—which stem from misfit dislocations—is a crucial factor influencing the transition. The decrease of the size of the scattering regions is a result rather than a parameter, influencing the final pressure-induced transition in SmS-based materials [43].

Both the transition and the resistivity change upon the transition can thus be influenced by substituting Sm with other elements. When substituting a large part of Sm by Eu, the transition is impaired in (Sm,Eu)S. Substitution of Sm by Eu (ionic radius of divalent Eu: 0.131 nm) leads to a shift of the pressure threshold for the semiconductor–metal transition to higher values. This behavior is accompanied by a shift of the lowest 5d band to higher energies. A different behavior is observed when Yb (ionic radii of divalent Yb: 0.116 nm) is used, as even a large amount of Yb induces only a relatively small reduction of the resistivity for the alloyed system, as the position of the 5d band remains relatively unaltered. Another case is found in the use of Ca^2+^ as alloying element, having an ionic radius (0.114 nm) comparable to Sm^2+^ (0.109 nm). This differs from the previous alloys, as CaS presents neither 5d conduction band nor 4f levels. Both the electronic structure and the size effect can however influence the switching and resistivity behavior of SmS [25]. In this case, the resistivity behaves similarly to the usage of EuS: for lower concentrations of Ca, a first order abrupt resistivity drop occurs, while for higher Ca concentration the system shows a continuous transition from the high to the low resistivity.

For alloyed SmS-based thin films, the mechanism behind the transition is explained as follows. In SmS, the system possesses its semiconducting phase under normal conditions. The substitution of additional elements into the structure of SmS such as Y or Eu promotes charge carriers into the 5d conduction band, with the lattice constant of the semiconducting SmS decreasing and approaching metallic values. After that, the transition takes place without any additional contribution, such as high pressure. Subsequently, as we can see in Figure 4a, the system collapses when the fraction of additional element in the structure reaches the critical concentration x_c_. Nonetheless, the multiplet structure of the 4f^6^ ground state of Sm^2+^ ion fully touches the Fermi level only at concentrations higher than x_c_. Overall, the transition in this kind of system takes place upon reaching the concentration x_c_, where the band gap between 4f and 5d vanishes [14,43].

The effect of Sm substitution on the SmS lattice parameter is dependent on the alloying element, and can be divided in three cases. When using Gd or Nd, as alloying elements, the lattice parameter shows a discontinuous decrease as a function of increasing Gd or Nd concentration. In the case of Yb and Ca, no discontinuous behavior is observed, while the usage of Eu (EuS shows almost the same lattice constant (0.596 nm) with S-SmS) does not lead to any change in lattice parameter over the whole range of concentrations.

**Figure 5:**
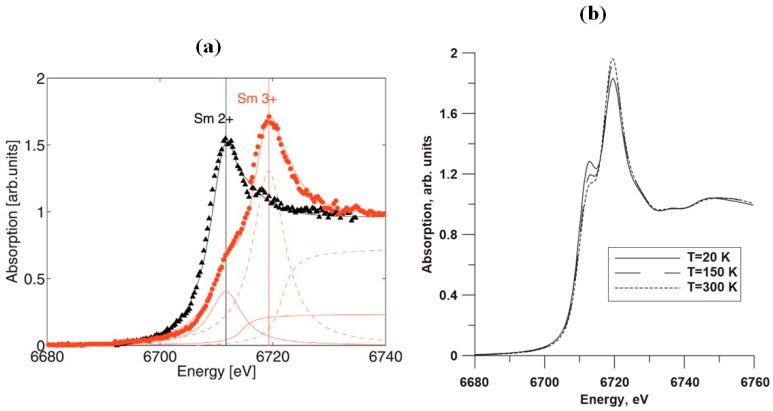
(**a**) X-ray absorption spectra at 4.5 K of the Sm^2+^ L3 transition of SmS. Black triangles correspond to a pressure of 0 GPa, while the red dots are upon application of 1.85 GPa; (**b**) XANES spectra of Sm_0.55_Y_0.45_S thin films at several temperatures, without application of pressure. (Reprinted with permission from [26] and [45], respectively.)

X-ray absorption near edge spectroscopy (XANES) results are shown in Figure 5a, where two XANES white lines are observed at about 6711.7 eV and 6719.3 eV, for divalent and trivalent Sm, respectively. At 4.5 K and 0 GPa (black triangles), only the divalent state of Sm was observed. By increasing the pressure to 1.85 GPa, corresponding to the golden phase, the white line for Sm^3+^ dominates, although a certain fraction of the Sm ions remains in the divalent state, which demonstrates the mixed-valence character [26,44]. In Figure 5b, we observe a similar diagram in the case of the Sm L3 XANES spectrum of Sm_0.55_Y_0.45_S, at several temperatures [45]. At lower concentrations of Y, the Sm^2+^ peak is stronger (not shown), and has about equal peak intensity in the case of Sm_0.67_Y_0.33_S. Increasing the temperature in both cases, the Sm^3+^ peak becomes dominant. Specifically, in the case of Sm_0.55_Y_0.45_S trivalent Sm becomes abundant. For these two concentrations, there is no sharp increase of lattice parameter, as the material acquires its semiconducting state, which is anticipated in the case of SmS. Possibly, the increase in temperature leads to an increase of Sm valence, with associated decrease of the lattice parameter, with presumably stronger 4f electron delocalization [45].

Several competing factors determine whether the semiconductor–metal transition is continuous or discontinuous [26]. The lattice contraction during the transition from Sm^2+^ to Sm^3+^ is counteracted by e.g., the higher bulk modulus at higher pressure (50 GPa at zero pressure, [28]) and the different density of states between the 4f and 5d bands. In addition, the 6s band has a crucial influence on the transition. If there is a strong hybridization between 5d and 6s states at the bottom of the conduction band, the transition will probably be continuous, due to the relatively low density of states at the Fermi level. If there is only 5d contribution to the bottom part of the conduction band, then a discontinuous or first-order transition is likely ([25] and papers therein).

By using X-ray photoelectron spectroscopy (XPS), the thermally induced transition between the two states, from the golden to the black one can also be observed [46]. XPS measurements have shown that both the semiconductor–metal transition and the oxidation process (a fraction of Sm oxide appearing after annealing) in SmS are responsible for the final change in color [47]. In another investigation, the changes in resistance were studied after pulsed laser irradiation at 308 nm, demonstrating that a fine modulation of the resistance of the black state of SmS can be achieved at lower fluences in comparison with the laser ablation fluence, without significant destruction of the films. The reason of this structural film modification is related to surface tension forces and thermally induced stresses [48]. In comparison with other work [49], these thin films presented less structural damage during transition.

### 2.4. Magnetic Properties

**Figure 6:**
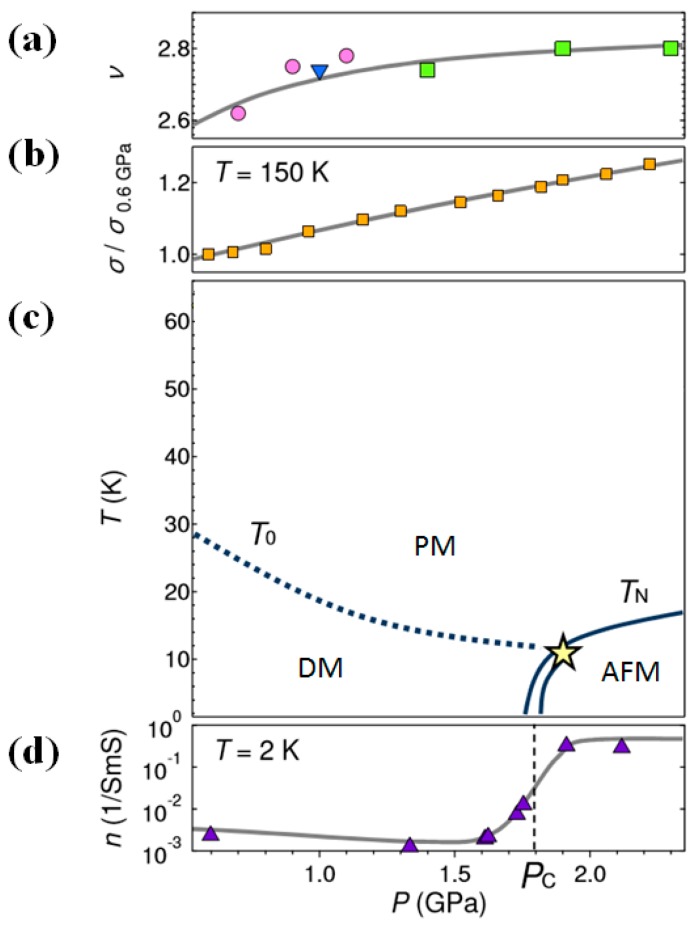
(**a**) Mean valence (already well above valence state 2) and (**b**) conductivity of SmS at the specific pressure interval between approximately 0.5 GPa and 2.4 GPa. Different shapes of (**a**) represent values coming from different works, cited in the original paper of Figure 6. Black line serves as a guide to the eye; (**c**) P-T phase diagram of the metallic phase, where PM: paramagnetic regime, AFM: antiferromagnetic regime and DM: diamagnetic regime; (**d**) Evolution of the number of free carriers (n) at T = 2 K. (Reprinted with permission from [50].)

SmS presents a pseudo-gap in the metallic state and shows paramagnetic behavior, while it is non-magnetic in the semiconducting state. The mean valence v (showing the number of electrons per Sm ion) and conductivity σ of SmS single crystal have a continuous monotonic increase with pressure at high T, as can be observed in Figure 6a,b. At low temperature, there is a critical pressure 1.8 GPa for the magnetic transition from the paramagnetic state at lower pressures to the antiferromagnetically ordered state at higher pressure. This is revealed by measuring the magnetic susceptibility (Figure 6c, yellow shapes). Consequently, a quantum phase transition occurs (at low temperature) at this pressure (P_c_ in the graph), which originates from the large variation in the number of carriers n at T = 2 K, as shown in Figure 6d ([50] and references therein). There are thus two kinds of transitions in SmS, a semiconductor–metal transition and a magnetic transition from the paramagnetic state to the antiferromagnetically ordered state.

In Figure 6c, T_0_ and T_N_ symbolize the temperature of the opening of a pseudo-gap and the Néel temperature, respectively. The star (tricritical point) marks the separation between a first order transition, indicated by the double line, and a second order transition (single line). Overall, there are two important aspects about the magnetic behavior of metallic SmS. Firstly, the Curie law provides a description of the localized moments at high temperature, with a pressure dependent ground state. In the case the pressure is lower than the critical one (1.8 GPa) and the temperature lower than T_0_, these localized moments disappear due to spin-singlet and exciton-like bound states. Secondly, by contrast, at a pressure greater than the critical pressure, the localized moments form an antiferromagnetic state, below T_N_ promoting the RKKY (Ruderman-Kittel-Kasuya-Yosida) interaction. This interaction stems from the coupling mechanism in a metal between its localized either f or d shell electron spins and nuclear magnetic moments with electrons of the conduction band via heavy quasiparticles. This means that there is a transition from a real bound state to a Kondo virtual bound state at this specific critical pressure [51]. It should be mentioned that this behavior is possibly related to a competition between antiferromagnetic ordering and pseudo-gap formation due to screening [40,51].

**Figure 7:**
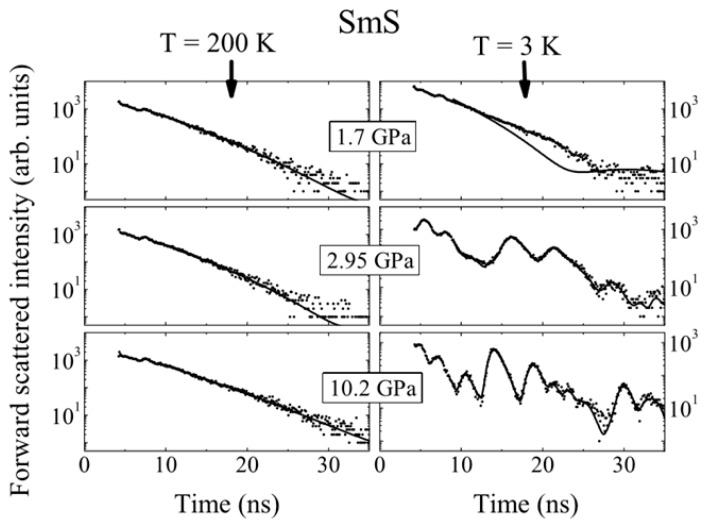
^149^Sm NFS spectra of SmS at different temperatures and pressures. Dots are the experimental results, while the full lines are fits. (Reprinted with permission from [52].)

The most important reason to study the magnetic properties of SmS is the instability between the magnetic states at the quantum critical point (QCP, at T = 10 K and P = 1.8 GPa). In this regime, phenomena such as unconventional superconductivity and the Kondo effect appear [13]. As mentioned, the Sm divalent state is non-magnetic, since the total magnetic moment is zero (J = 0), as L = S = 3, while Sm^3+^ shows magnetic behavior. Barla et al. [52] studied the SmS magnetic properties by measuring Nuclear Forward Scattering (NFS) spectra at various temperatures and pressures of metallic SmS (Figure 7). By using this technique, the quantum beat pattern was obtained from the combined action of magnetic dipole and electric quadrupole interactions on the nuclear level of the isotope ^149^Sm [52]. This behavior seems to exist only at low temperatures. The reason that no effect was observed at higher temperatures (for a variety of pressures) is related to a common behavior of unsplit nuclear levels for Sm ions in the absence of magnetic order within a cubic symmetry environment [52]. It was shown that only part of the Sm atoms, corresponding to 72%, at 2.35 GPa and 3 K showed a magnetic order, the remainder being paramagnetic. This amount (28%, paramagnetic) was transferred to its final magnetic state at 3 GPa.

At temperatures and pressures lower than T_0_ and P_c_, the ground state of metallic SmS is diamagnetic, as the magnetic susceptibility does not demonstrate any paramagnetic Curie–Weiss divergence [53]. Several studies [13,50] have reported the temperature dependence of the thermal expansion coefficient α(T) of metallic SmS. All these measurements took place by using SmS single crystals. NFS and thermal expansion experiments show a tricritical point (T_c_), as we see in Figure 6c (star symbol), where the separation of the first order Néel transition from the second order transition takes place. By combining all these results, a P-T diagram can be obtained similar to Figure 6c, consisting of the first and second order lines due to the Néel temperature, and the crossover line stemming from T_0_.

**Figure 8:**
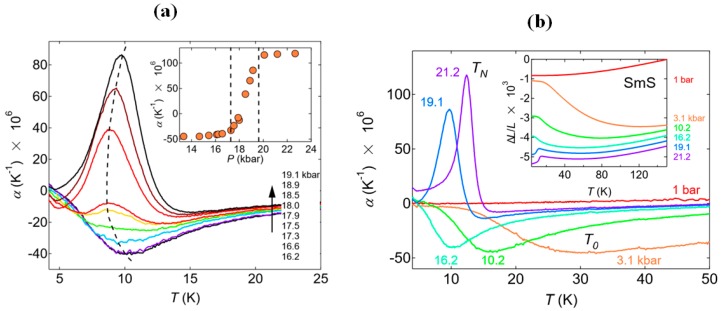
(**a**) Temperature dependence of the thermal expansion coefficient at pressures up to 21.2 kbar (2.12 GPa), with T_0_ and T_N_ corresponding to shallow negative and sharp positive peaks, respectively and a ΔL/L(T) graph in the inset. Data have been shifted by the authors for clarity; (**b**) A similar graph at pressures between 16.2 and 19.1 kbar (1.62–1.91 GPa). The area which is created by the two vertical dashed lines in the inset demonstrates the mixed phase regime. (Reprinted with permission from [13].)

There are two main characteristic peaks in the temperature dependence of the thermal expansion coefficient. A shallow minimum and positive sharp peaks are seen in Figure 8a, which are related to T_0_ and the Néel temperature, respectively. The positive peak at pressures above the second critical pressure can be understood from the inset graph ΔL/L(T) as well, where we see a strong change at these pressures [13]. In Figure 8b, the evolution of the thermal expansion coefficient for a pressure interval between 16.2 and 19.1 kbar (1.62 and 1.91 GPa) is shown. The shallow minimum at 10 K for low pressure changes to a sharp positive peak upon increasing the pressure. This describes the mixing of the two magnetic phases, which consists of a broad minimum and a positive peak stemming from the paramagnetic and antiferromagnetic states, respectively. The temperature dependence of the compressibility shows a similar behavior: broader peaks at lower pressure and narrower peaks close to T_N_, which is consistent with the behavior of α(T). Measuring magnetic susceptibility χ and the pressure dependence of the Hall coefficient R_H_ is another way to obtain T_N_ [36,54]. In pressure dependent measurements of R_H_ of the metallic state of SmS, there is a transition from values above 0 to values below 0 at a pressure of about 1.75 GPa at constant temperature 2 K, which describes the magnetic transition. Exploiting all these kinds of anomalies, a P-T graph similar to that of the Figure 6c can be made.

In the previous part, we analyzed results from optoelectronic and magnetic properties of SmS. In this part, we will discuss more exotic properties of this material, such as electrical oscillations and Kondo insulator behavior. A very recent investigation about electrical oscillations in SmS was reported by Takahashi et al. [55]. Another way to induce the semiconducting–metallic transition, apart from using pressure, is by applying a DC electric field (on the order of a few kV/cm), inducing a collective motion of charges causing an electrical oscillation [55]. At high electric fields (4 × 10^3^ V/cm), nonlinear conduction phenomena are observed. Electrical oscillations are an additional effect, which takes place during the semiconducting–metallic transition and it has been observed in several materials, such as correlated insulators [56,57]. In the case of the semiconducting SmS, a persistent electrical oscillation has been observed by applying constant external voltage (DC). In general, the f-electron systems show prominent correlation behavior, which is the main reason of the aforementioned phenomenon, which is very similar to the tunneling mechanism and negative differential resistance in narrow gap semiconductors (Zener effect) [55].

It is still a point of discussion whether a Kondo insulating behavior is present in SmS. A Kondo insulator is a material for which the electrical resistivity dramatically increases—a narrow band gap opens—at low temperatures. This is a result of the hybridization of localized electrons with electrons of the conduction band. Previously, we have noted the existence of a mixed valence state and a pseudo-gap in SmS. The excitonic instability is a factor that determines the pseudo-gap, but, in another work, the hybridization of the 4f band into 4f_5/2_ and 4f_7/2_ has also been mentioned as an important parameter. The 4f_5/2_ band is placed quite close to the Fermi energy, whilst the 4f_7/2_ level is located about ±0.17 eV away from the Fermi energy [51]. Kondo behavior stems from strongly correlated 4f systems, so SmS is a possible candidate, as described by Li et al. [58]. However, Kang et al. [51] proposed a different model for SmS instead of that of a conventional Kondo insulator. In that case, the behavior of SmS has been analyzed by using dynamical mean-field theory (DMFT), properly describing the insulating ground state of SmS and the topological properties of the SmS metallic state of strongly correlated 4f electrons. In order to study the topological properties of metallic SmS, a surface band structure has been calculated (not shown). A tiny gap was found, which demonstrates that Rashba spin-polarized surface states (i.e., a momentum dependent splitting of spin states) play a role instead of topologically protected Kondo states with a Dirac cone in the band structure. Nonetheless, elsewhere [59], Sm_0.75_La_0.25_S was found to have an excitonic insulating gap equal to 1 meV, describing it as a possible Kondo insulator. Since then, SmS has been characterized as a Kondo semimetal, instead of a Kondo insulator, like the very similar compound SmB_6_ (showing an intermediate valence at ambient pressure) [23,60].

## 3. Thin Film and Bulk SmS Preparation Techniques

### 3.1. Electron Beam Evaporation—Reactive Evaporation

Electron beam evaporation (EBE) is a relatively cheap and flexible method for the deposition of a wide range of compound thin films. Apart from the choice of evaporation source, deposition speed and substrate temperature, the film properties and stoichiometry can be influenced to some extent by reactive evaporation. In that case, a reactive gas is introduced in the vacuum system, with a low pressure, limited by the maximum working pressure of the e-beam filament. The e-beam deposition of SmS is often started from a Sm metal source, accompanied by an H_2_S flow. Rogers et al. described the deposition of homogeneous thin films by using e-beam evaporation and appropriately balancing the Sm evaporation rate and the H_2_S partial pressure, to arrive at the desired stoichiometry. A continuous semiconductor–metal transition was observed from optical measurements, but simultaneously a discontinuous transition was taking place on a grain to grain basis, similar to results published elsewhere [15,61]. The XRD peaks pointed at a randomly oriented structure in comparison with thin films deposited using sputtering techniques, where strongly textured thin films can be obtained [15,61]. Another similar investigation based on e-beam evaporation comes from Hickey et al., who used several types of substrates, such as PMMA, quartz and soda lime glass. The main characteristic was the presence of a relatively high level of impurities, such as oxygen [12,41].

### 3.2. Sputtering

**Figure 9:**
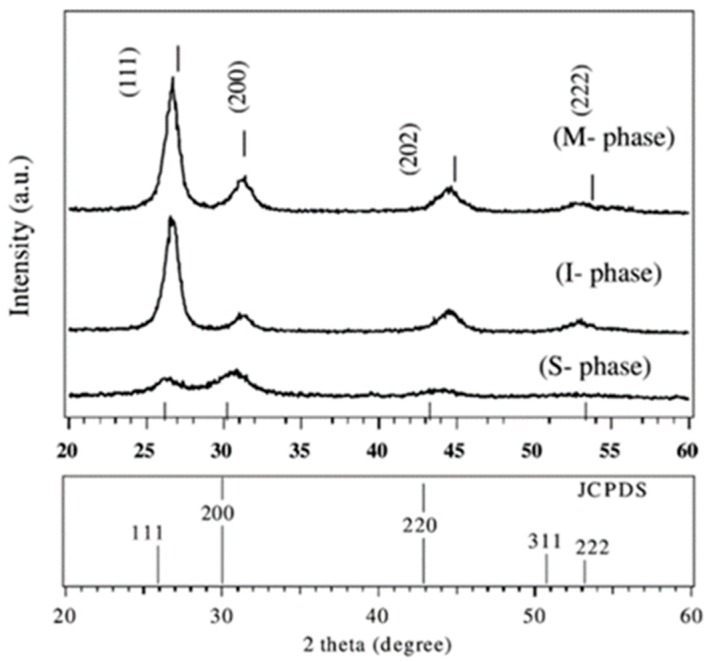
XRD patterns demonstrating the three possible states (semiconducting–intermediate–metallic) and a pattern with the standard diffraction peaks of SmS. (Adapted from [63], with permission.)

Sputtering, in both RF and DC operation, has been used for the deposition of SmS [62,63]. Given that a SmS target is difficult to obtain, one can combine DC magnetron sputtering using a Sm (metallic) target and an additional RF magnetron with a chalcogenide (Sm_2_S_3_) target to eventually obtain SmS. The structural properties of the resulting films were determined using XRD. Three possible phases could be obtained depending on the ratio P_rf_/P_dc_ (see Figure 9). For example, the metallic phase was obtained for a ratio equal to 2, a quasi-metallic state (intermediate state) for a ratio equal to 3.2 and the final semiconducting phase was obtained for a power ratio of 5.46.

In another investigation [61], by properly changing the substrate temperature and the power of the Sm and Sm_2_S_3_ RF sputter targets (and thus their deposition rates), different phases of Sm*_x_*S (*x* is the ratio of Sm to S) could be deposited, without any trace of SmO. Possibly, the most intriguing result of this work is that for *x* > 1, the metallic phase of SmS was obtained as-deposited. In all cases of deposition power and substrate temperature, the most prominent diffraction peak was that of (200). Nonetheless, there was a splitting of this peak into two peaks at higher deposition temperatures, especially at 275 °C, indicating the simultaneous presence of the semiconducting and metallic phase. This was mainly observed (away from *x* = 1) in the case of *x* = 3.8 and *x* = 2.3. When *x* decreased below one, new phases were created and the sulfur-rich phases Sm_3_S_4_ and Sm_2_S_3_ were formed. Values of *x* close to one yielded a semiconducting SmS with corresponding lattice constant. Generally, it is evident that one of the advantages of using a co-sputtering technique with two targets is that by adjusting the parameters of sputtering there is a possibility to manipulate the dominating phase, semiconducting or metallic, in as-deposited thin films [61,62,63].

### 3.3. Pulsed Laser Deposition

**Figure 10:**
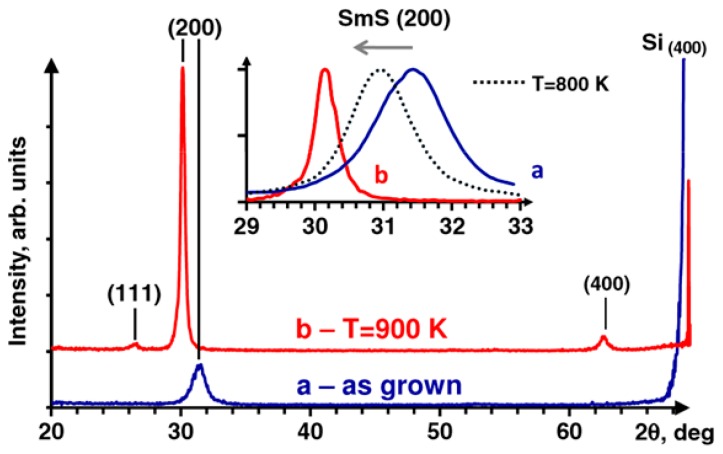
X-ray patterns of SmS thin films deposited using PLD. The blue curve shows the as-deposited SmS at room temperature, which presents a metallic behavior with low intensity of the Bragg reflection. This behavior was also supported by electrical and optical measurements. The red curve represents the Bragg reflection of annealed film at 900 K, in vacuum. The inset details the shape of the (200) peak for the two cases and for an annealing at 800 K (grey dotted line). (Reprinted with permission from [64].)

For pulsed laser deposition (PLD), the energetic species in the condensing flux can provide stress modification during the thin film growth. Starting from a SmS target, Zenkevich et al. manufactured highly oriented semiconducting SmS on Si (100) at elevated substrate temperature, between 600 and 1000 K [64]. In Figure 10, we see that there are no other main reflections apart from (200), with a corresponding lattice parameter a = 0.569 nm and an estimated grain size of 8 nm (blue line). Nevertheless, the grain size, as calculated from XRD peak broadening, could be misinterpreted; the presence of stress would also increase the FWHM of the XRD peaks. An increase in the annealing temperature shifted the XRD peaks to smaller angles. In the case of film annealed at T = 900 K, a lattice parameter a = 0.593 nm was obtained, corresponding to the semiconducting phase of SmS.

### 3.4. MOCVD of SmS

**Figure 11:**
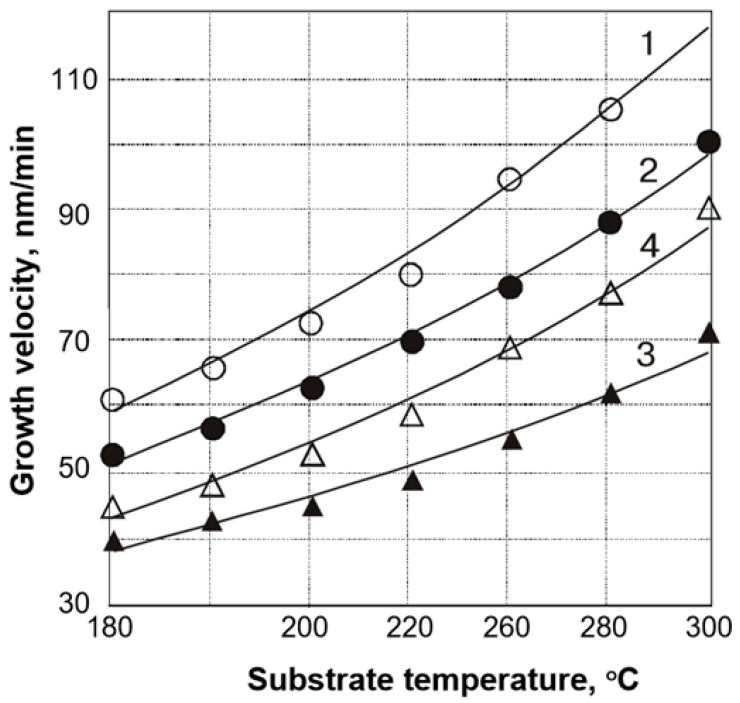
Dependence of MOCVD growth velocity of SmS for different substrate temperatures and the used precursors: 1, dtc3Sm bipy; 2, dtc3Sm phen; 3, dtc3Sm (without water ambient); and 4, dtc3Sm (with water ambient). (Reprinted with permission from [65].)

Volodin et al. [65] described Molecular Organometallic Chemical Vapor Deposition (MOCVD) of polycrystalline SmS. In MOCVD one of the most important parameters is the choice of precursors. The following initial substances have been used in the case of SmS. Diethyldithiocarbamat (DTC) of samarium (dtc3Sm), dtc3Sm phen, which additionally contains phenantraline ligands and dtc3Sm bipy, which additionally contains pyridil ligands. The MOCVD synthesis of SmS is described in detail by Stern et al. [60]. The best results were obtained with dtc3Sm bipy, providing maximum growth rate, polycrystallinity, continuity and uniformity of the SmS thin films. Figure 11 shows the behavior of the growth velocity versus the substrate temperature for four precursors used. Using higher amounts of ligands led to an acceleration of the growth (lines 1 and 2). As anticipated, the growth rate increased at higher temperature. Nonetheless, as we can see in lines 3 and 4, there was a lower growth rate when using a waterless ambient (line 3) compared to one containing water (line 4), which was related to the purity of the dtc3Sm used. The final structure of the SmS films strongly depended on parameters such as temperature and substrate material, as well as the precursors. In general, all of these as-deposited MOCVD SmS thin films showed lattice parameters close to that of the metallic (or intermediate) state of about 0.57 nm.

### 3.5. Other Thin Film Deposition Techniques

In addition, molecular beam epitaxy (MBE) and electrodeposition have been used for the deposition of SmS. In the case of MBE, both samarium and sulfur were vaporized on a heated substrate at 450 °C. Either SmS or Sm_2_S_3_ thin films could be obtained by properly changing the sulfur flux [66].

Electrodeposition was used to obtain SmS thin films on ITO coated substrates through an aqueous solution which consisted of SmCl_3_·6H_2_O and Na_2_S_2_O_3_·5H_2_O. The final phase of SmS was semiconducting, but there was a fraction of Sm_2_S_3_ in the films, as indicated by XRD measurements. Annealing took place at 200 °C for 30 min, leading to crystallites with both elliptic and circular shape [67].

### 3.6. Bulk—Single Crystals

While metallic SmS thin films do not switch back to the semiconducting state upon releasing the pressure, the switching is reversible in single crystals. In the literature, several investigations have been published on the growth of single crystal SmS following mainly the same procedure [24,68,69,70,71], as proposed by Matsubayashi et al. Initially, they prepared the starting materials (Sm chips and powdered S) placing them in an evacuated quartz ampoule at 600 °C for several days. Afterwards, they used an electron beam welding system in which the starting materials (in a tungsten crucible) were fused. Subsequently, this step was followed by a vertical Bridgman growth in a high frequency induction oven [68]. The starting material for the Bridgman growth was placed in a tungsten crucible and heated up to 2180 °C (heating rate: 55 °C/h) for 21 h. After that, the sample was cooled down at a rate of 10 °C/h to 1900 °C, obtaining the SmS crystal in the semiconducting phase.

## 4. Applications

In this section, we present several applications based on the switching and thermoelectric behavior of SmS. Apart from highlighting the opportunities, we also discuss existing practical difficulties for the final applications. The main reason why the number of real-world applications of the switching properties of SmS is still limited, lies is the necessity for high quality single phase SmS films with the correct stoichiometry and Sm valence state. Form the discussion on the different thin film deposition techniques above, it is clear that SmS cannot be straightforwardly obtained, and more research is required in the field.

**Figure 12:**
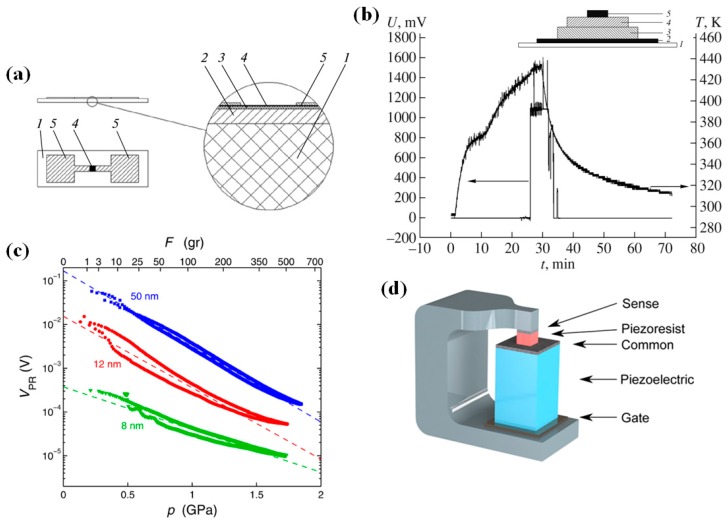
(**a**) A detailed form of a SmS-based strain gauge sensor (see text for the different parts); (**b**) Structure of a thermoelectric generator and behavior of voltages and temperature as a function of time; (**c**) Voltage (at 1 μA constant current) of SmSe for three different thin film thicknesses. Results were carried out using an indenter (top axis; load in grams) and finite elements calculations (bottom axis); (**d**) Schematic illustration of the proposed PET device. (Reprinted with permission from [72], [77] and [80], respectively.)

A quite mature application is that of a strain gauge, where SmS could be used, even if in this case there is no direct exploitation of the switching properties of the material. The change in electrical resistivity upon application of stress was monitored by using metallic thin films made by sputtering, as they have shown a strong strain-resistivity relationship. The existence of impurities can influence the pressure-induced resistivity drop of SmS, even in the metallic state [24]. Unfortunately, SmS is rather fragile under stress, and stress overloading can compromise the lifetime of the gauge (with piezoresistive gauge −2.0 GPa^−1^) [24]. A typical structure of a semiconductor strain gauge based on SmS is seen in Figure 12a. The substrate of this device is a lacquer (1), where on top of it a dumbbell shaped constantan carrier (2) is deposited. After that, a SiO (Silicon Monoxide) layer (3) is deposited onto surface 2, as a dielectric layer, with SmS (4) to be the next part of this device. Finally, a nickel layer (5) plays the role of contact [72].

One of the most intriguing applications using the switching behavior of SmS is in the application area of optical storage. If small spots on a SmS surface can be switched in a controlled way, this could be used to store and read information. It was shown that for suitable semiconducting spots (placed on a metallic surface/substrate) with a diameter on the order of 0.5 μm and by using laser pulse energies of 0.5 nJ/(μm)^2^, a potential data density of about 50 MB/cm^2^ can be achieved. The metallic bulk phase could be returned back to the semiconducting phase by local annealing with a pulsed laser. Nevertheless, the shock wave of the laser beam can reconvert the material back to the metallic phase, resulting in a decrease of the density of the optical data storage by a factor of 10 with a simultaneous increase of the required writing energy and the area between the spots. A similar study has been performed by Petrov et al. on holographic data storage, by using nano- and pico-second laser pulses [73]. Unfortunately, technical difficulties appeared. When an intense laser pulse was used, a written spot could be deleted.

There is a category of optical storage materials, named phase change materials, which have been studied in previous years, providing important results. Specifically, using these materials, around 2 Gbit/cm^2^ data storage density can be achieved in optical storage media (Blue Ray disc), a capacity which is still magnitudes higher than what can be obtained in SmS. As a result, for improving the specifications of SmS-based memories, both materials and device improvements should take place [48,74,75,76].

Another possible application of SmS is as thermoelectric generator. This application is possible thanks to an emerging voltage across a heated SmS bulk material, without the presence of external temperature gradients. This can be observed in the temperature interval from 400 to 500 K. In order to maximize the voltage, a maximum concentration gradient of Sm ions is necessary. A basic structure which has been manufactured, consisted of a Al_2_O_3_ substrate (1), a metallic contact made from nickel (2), on which an overstoichiometric semiconducting Sm_1.1_S thin film (3) was subsequently deposited, in the next step an additional semiconducting SmS thin film (4) and finally a metallic contact of Ni (5). When the temperature had reached 428 K an electric voltage equal to 1.1 V appeared, but when the temperature decreased to 360 K, the voltage disappeared (Figure 12b) [77]. In a very similar investigation (on bulk SmS), the specific voltage generation due to the thermoelectric effect could be seen between 100 and 1800 K [78]. Actually, the screening of the Coulomb potential of the additional Sm ions by the electrons of the conduction band plays an important role in the occurrence of the thermoelectric effect, as at higher temperatures there is an activation of electrons coming from the 4f Sm ion level to the conduction band [78]. Recently, it was described that γ-SmGdEuS_4_ shows a p-type Seebeck coefficient and high electrical resistivity, providing opportunities for thermoelectric applications in this class of materials [79].

The next step is the manufacturing of ultrathin films, which still demonstrate switching behavior on the nanoscale. This would open the way to several applications, with non-volatile electrical storage memories as the most important one. This has been confirmed by very recent results on the piezoresistive behavior of SmSe [80], which could be extended to SmS, promoting a new low-energy memory device, which is based on piezoelectric (PE) and piezoresistive (PR) components. There are three conductive parts (gate, common, sense) in this device. The PE material, which is placed between the gate and common, expands upon application of a voltage, triggering a semiconductor–metal transition in the PR layer that is placed between the common and sense. All of these components are mounted in a yoke, which has a high Young modulus (Figure 12d). In Figure 12c, we see the voltage (proportional to resistance) of three SmSe thin films as a function of applied pressure. The top axis represents the load of an indenter (in grams), while the bottom axis shows the corresponding pressure from calculations (finite element calculations; in GPa). The change in voltage is clearly seen, by applying pressure [80]. In this kind of application, SmS can provide a hysteretic behavior, which does not take place in the case of SmSe. This would decrease at the same time the required input voltage, which is responsible for the expansion of the PE part of the device and as a result for compression of the PR material, which will change between a low and a high resistance state (metal–insulator transition) translating these changes to 0 and 1. In this case, it is a challenge to modify the composition in such a way that the transition back to the semiconducting state can be achieved without annealing in vacuum. Alloying with other elements, such as EuS and GdS can be used to induce a repeatable, reversible switching between the states. This idea can promote an appropriate hysteretic resistivity loop as a function of pressure, provided clamping effects of substrate and other components are properly taken into account.

## 5. Conclusions

In this review, material properties and possible applications of samarium sulfide were reviewed. SmS is a switchable material, which shows a first order transition (semiconductor–metal) at about 0.65 GPa, characterized by a volume collapse without a change in crystallographic phase, and an additional magnetic transition at about 1.9 GPa. SmS presents many intriguing phenomena such as Kondo-like behavior and the existence of a pseudo-gap before the final closing of its band gap, when reaching the metallic phase. Different experimental and computational techniques were used to study its switchable properties, giving at the same time additional information about practical applications. A crucial issue is the presence of the suitable oxidation state of Sm, which is responsible for the switching behavior. We have to note that SmS bulk crystals properties have been more thoroughly studied in comparison with SmS thin films, although for thin films the influence of stress, induced by the substrate, can strongly alter the switching behavior. Obtaining the correct stoichiometry in SmS can be an experimental challenge, although several techniques are described in literature, such as pulsed laser deposition, electron beam evaporation and sputtering. By alloying, for instance by europium or gadolinium, the resistivity and the switching behavior can be altered. While the switching in SmS was initially dominantly studied from a fundamental point of view, the first application interests were in strain sensing and optical memory devices, as, in the latter case, laser irradiation can also induce the switching. Recently, non-volatile memory devices based on combined piezoelectric and piezoresistive materials have been proposed. In summary, SmS can be considered as an intriguing switchable material, as it shows a pressure-induced semiconductor–metal transition at moderate pressure, at room temperature. Nevertheless, the need for high-quality SmS thin films in view of applications simultaneously boosts the study of fundamental properties of SmS thin films. This will allow the usage of this material in high-quality sensing and memory devices, as well as very presumably in optical switching devices, due to the laser-induced transition of SmS. These applications will promote a further study not only of SmS thin films, but also of other pressure-induced insulator–metal compounds.
